# Comparative Evaluation of Metal Ions Release from Titanium and Ti-6Al-7Nb into Bio-Fluids

**Published:** 2009

**Authors:** Lori A. Joseph, Omoniyi K. Israel, Ekanem J. Edet

**Affiliations:** * PhD of Chemistry, Department of Chemistry, Ahmadu Bello University, Zaria, Nigeria; ** MSc in Chemistry, School of Basic and Remedial Studies, Ahmadu Bello University, Funtua, Nigeria

**Keywords:** Biocompatibility, corrosion, ions, pH, titanium

## Abstract

**Background:**

The study was designed to investigate the effects of pH, chloride ions and nature of some bio-fluids on the amount of metal ions released from titanium and TiAl_6_Nb_7_ plates following incubation in actual and simulated bio-fluids over time.

**Methods:**

The amounts of released metal ions from commercially pure titanium (CpTi) and TiAl_6_Nb_7_of surgical grade on immersion in 20 mL Hank’s solution of pH 4.0 or 7.0, Hank’s solution of high chloride ions concentration, Whole Blood Serum (WBS) and Phosphate Buffered Saline (PBS) at 37° C were determined over an incubation time of 20 weeks using atomic absorption spectrophotometry. The levels of released metal ions were compared by two-way ANOVA and Duncan’s post-hoc tests. The amounts of titanium ions released by the samples were analyzed by Pearson’s correlation.

**Results:**

TiAl_6_Nb_7_ plate showed no release of Ti ions into the test solutions until after 12 weeks of incubation, while Ti ions were released from the CpTi plate from the 1 day immersion time. The release of measurable amount of Al ions from TiAl_6_Nb_7_was after 12 weeks of incubation. The rate of release of Ti and Al ions from the samples increased initially with incubation time and then stabilized due to adsorption-desorption equilibrium.

**Conclusion:**

The results showed that variations in pH and chloride ions of the test media has a significant effect on the amounts of Ti ions released, while increase in chloride ions concentration significantly elevates the release of Al ions into the biofluids.

## Introduction

Titanium (Ti) has been widely used in biomaterial fields.^1^ Its high inertia due to the formation of a thin surface titanium oxide layer, light weight and excellent biocompatibility make it the ideal material for surgical implants.^2^ The problems with poor mechanical strength of pure Ti were overcome by the addition of 6% aluminum and 4% vanadium to commercially pure Ti resulting in an alloy (TiAl_6_V_4_) with mechanical properties similar to those of stainless steel or Co-Cr alloys. Recent reports of the release of small amounts of aluminum (Al) and vanadium (V) into the human body - both being toxic to human body - has resulted to the Ti based alloy, (TiAl_6_Nb_7_) where V is substituted by niobium (Nb) and another (TiZr_15_Nb_4_Ta_4_) where V is substituted by niobium (Nb) or tantalum (Ta) and Al is substituted by zirconium (Zr). The pure Ti and Ti based alloys have inherent corrosion resistance attributed to the spontaneous formation of a strong passivating oxide layer. The extent of this corrosion resistance dictates their biocompatibility, useful lifetime and often their functional ability.^3^

When metallic implants are placed in the electrolytic environment provided by the human body, the electrochemical reaction resulting to the release of metal ions is coupled with a corresponding reduction reaction of constituents in the aqueous environment to maintain charge neutrality.^4^ How much of these metal ions are released is a function of the corrosion resistance of the metal, the physiological conditions (pH, chloride ion concentration, temperature, etc.), mechanical factors (pre-existing cracks, surface abrasion and film adhesion), electrochemical effects (galvanic effects, pitting or crevices) and the dense cell concentrations around implants.^5^

Metal ions generated by implants and their wear debris may remain in local tissues while some may bind to protein moieties that are then transported through the blood stream and lymphatics to remote organs. Although toxic effects of Ti, Al, V, Co, Cr and Ni in implant alloys have being reported, toxicology generally applies to soluble forms of these elements and may not apply to the chemical species that result from degradation of prosthetic implants.^6^

Ferguson et al were the first to document locally elevated titanium levels in the presence of a titanium implant.^7^ Relation between the fluoride concentrations and pH of corrosion environments on Ti ions leached from implants was investigated.^8^ Standardized artificial implant alloys (TiAl_6_V_4_, Ti, CoCr_29_ Mo and FeCrNiMoMnNbN) were immersed in inert polystyrene test tubes filled with 1 mL of serum (pH 7.4). The dissolution of the metal ions depended on the proportional mix of the individual elements in the respective alloys. The highest percentage of ion concentration was found for Co in CoCr_29_ Mo and the low percentages of ion concentration were evident for Ti (Ti), Cr (CoCr_29_Mo) and Cr (FeCrNiMoMnNbN).^9^

This study aimed to investigate the effects of pH, chloride ions and nature of some bathing biofluids on the amount of metal ions released from Ti and TiAl_6_Nb_7_ plates following incubation in actual and simulated biofluids over time. A comparative assessment of the release of metal ions from Ti and its alloys is significant in elucidating the longterm biocompatibility profile resulting from alloying of the test materials.

## Materials and Methods

Commercially pure titanium (CpTi) and TiAl_6_Nb_7_plates (KS-50, Kobe Steel, Kobe, Japan) of surface area, 10mm × 10mm × 1 mm, were used as the test materials.

### 

#### Bio-fluids for the study

Whole blood serum (WBS) was obtained by collecting fresh bovine blood of a healthy male cow into slantly positioned boiling tubes. WBS obtained after about 3 hours had a pH of 7.68.

1. Hank’s solution (HS), to simulate body fluid was prepared with the following ionic composition (mol L^−1^) : Na^+^ 1.42 X 10^−1^ ; K^+^ 5.81 X 10^−3^ ; Mg^2+^ 8.11 X 10^−4^ ; Ca^2+^ 1.26 X 10^−3^ ; Cl^−^ 1.45 X 10^−1^ ; HPO_4_^2−^ 7.78 X 10^−4^ ; SO_4_ ^2−^ 8.11 X 10^−4^ and CO_3_^2−^ 4.17 × 10^−3^.^10^
2. The solution was adjusted to pH 4.0 (HSpH4) and pH 7.0 (HSpH7) with 0.5M Na_2_HPO_4_and 0.5M NaH_2_PO_4_ buffer solutions and drops of 1% HCl solution. The concentration of HS with high chloride ions (HSCl) was obtained by making the concentration of chloride ions in HS to be 1.63 X 10^−1^ molL^−1.^
3. Phosphate buffered saline (PBS) solution (Nissui Pharmaceutical Co. Ltd., Tokyo, Japan) with a pH of 7.45.

#### Invitro corrosion study

Corrosion was monitored by the amount of metal ions measured in the incubation biofluids. A plate of CpTi or TiAl_6_Nb_7_was immersed in inert poly-propylene bottle filled with 20 mL of Hank’s solution, HS at pH 4.0, pH 7.0 or HS with high chloride ion (HSCl), the container was closed and incubated at 37°C. Three independent samples were prepared for each experimental group. Following incubation at the end of the 1 day, 1, 2, 4, 8, 12 and 20 weeks, 10 mL of each of the experimental solutions were withdrawn with a syringe after shaking. These were then digested with 3 mL of concentrated HNO_3_ and 10 mL of concentrated HCl solution on a hot plate at 100^°^ C. The digest was then made up to 20 mL with doubly distilled water.^11^ The digestion was repeated for each of the experimental biofluids. These corrosion experiments were also performed for WBS and PBS.

The concentrations of Ti ions released from CpTi and also Ti and Al ions released from TiAl_6_Nb_7_plates into the three mentioned bio-fluids were measured using a graphite furnace atomic absorption spectrophotometer (model TAS990, Intec Co. Ltd., Rome, Italy). The concentrations of these ions in the bio-fluids prior to incubation were taken as control. The quality assurance for the analyses was conducted through the spiking method, and mean percentages of recovery were calculated. The levels of released metal ions were compared by two-way ANOVA and Duncan’s post-hoc tests (p < 0.05) and the amounts of titanium ions released by the samples were related by Pearson’s correlation.

## Results

**Figure 1:**
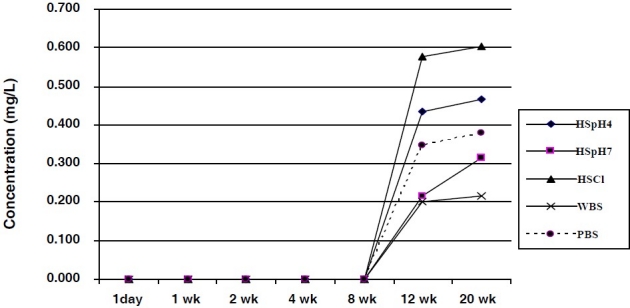
Titanium ions released from Ti-6Al-7Nb into bio-fluids

**Figure 2:**
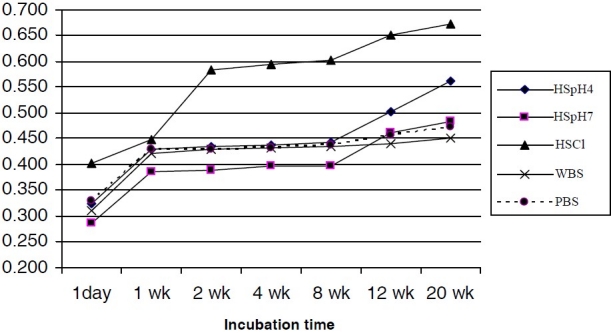
Titanium ions released from CpTi into bio-fluids

The results are expressed as the average of triplicate determinations ± the standard deviation and presented in Figure 1–2. Data was analyzed using statistical software (SAS), and a two-way ANOVA and Duncan’s Post-hoc multiple range tests (DMRT) were used to assess differences in the mean values of the metal ions released due to effects of media and incubation time of the test materials in the biofluids. Pearson’s correlation was used to find whether or not relationships exist between the amount of metal ions released by the two test materials at p < 0.05 levels of significance.

## Discussion

### 

#### Release of Ti ions

The results of this study showed that there was no detectable amount of released Ti ions from the TiAl_6_Nb_7_samples into the various bio-fluids until after 12 weeks of incubation. The highest concentrations were measured in HSCl, with values 0.578 ± 0.001 mg/L after 8 weeks and 0.603 ± 0.002 mg/L after 12 weeks of incubation. The rate of release of Ti ions from the Ti alloy ranged from 0.0167 ± 0.001 to 0.0503 ± 0.003 μg/mL/week over the incubation time. The detection limit was 0.04μg/mL (Figure 1). DMRT showed a significant difference in the amount of Ti ions released into the various media, increase in chloride ions showed a strong difference and decrease in pH a less significant difference in the amounts of Ti ions compared to the other media. A statistically significant difference in the amount of Ti ions released from the sample was recorded beyond 8 weeks of incubation.

On the other hand, as shown in Figure 2, CpTi released measurable amount of Ti ions into the various bio-fluids from the 1 day incubation time. The chloride rich pseudo-body fluid (HSCl) and WBS resulted in significant release of higher mean concentration of Ti ions from CpTi compared to the other test solutions. There was no statistically significant difference between the amount of Ti ions released due to the effect of incubation time. The Ti ion release rate reaches a maximum after 1 week and then the rate of release diminishes with time. The rate of release of Ti ions from CpTi samples ranged from 0.023 ± 0.002 to 0.448 ± 0.004 μg/mL/week. The ranking from mild to aggressive corrosion media for the bio-fluids was HSpH7 < WBS < PBS< HSpH4 < HSCl. There was significant difference between the amounts of Ti ions released by the CpTi sample compared to that released by TiAl_6_Nb7. Pearson’s correlation depicted a positive relationship in the mean amount of Ti ions released into the bio-fluids by CpTi and TiAl_6_Nb_7_ (r = 0.889), with a highly statistical level of significance (p < 0.05).

#### Release of Al ions

**Figure 3:**
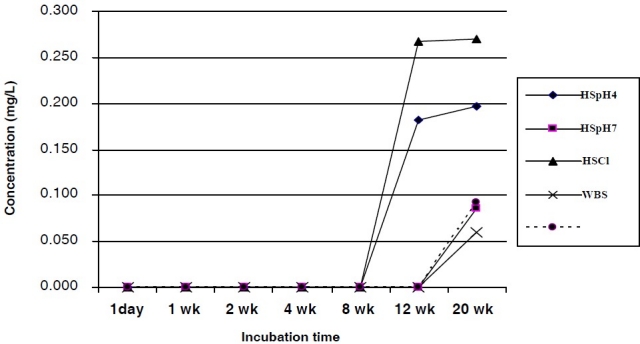
Aluminum ions released from Ti-6Al-7Nb into bio-fluids

The release of measurable amount of Al ions into the pseudo-body fluids were recorded after 8 weeks of incubation for HSCl and HSpH4 and after 12 weeks for PBS, WBS and HSpH7 as presented in Figure 3. The amounts of released Al ions into the five corrosion media after 20 weeks of incubation ranged from 0.060 ± 0.002 to 0.270 ± 0.004 mg/L. The detection limit was 0.02 μg/mL. DMRT showed a significant difference in the amount of Al ions released into the chloride rich environment (with release rate of 0.014 ± 0.001 μg/mL/week) compared to the other media, for instance, HSpH7 with Al ions release rate of 0.007 ± 0.001 μg/mL/week. Change in pH of the fluid-bio showed no statistical significance in the amounts of Al ions released, likewise extending the incubation time, until after 12 weeks. Pearson’s correlation showed a positive relationship in the mean amount of Al and Ti ions released into the bio-fluids by TiAl_6_Nb_7_ (r = 0.829), but it was not significant.

This result conforms with that of Okazaki and Gotoh (2002) where the quantities of Ti ions released from Ti-15Zr-4Nb-4Ta, Ti-6Al-4V and Ti- 6Al-7Nb alloys into phosphate buffered saline, α- medium and fetal bovine serum were very small compared to that in more acidic L-cysteine, 0.05 mass % HCl and 1 mass % lactic acid solutions. 12

## Conclusion

In this study TiAl_6_Nb_7_ plate showed a remarkable resistance to the release of Ti ions into the test solutions until after 12 weeks of incubation compared to CpTi and this can be due to the presence of 6% Al and 7% Nb in the material. The rate of release of Ti ions from the Ti alloy ranged from 0.017 ± 0.001 to 0.050 ± 0.003 μg/mL/week. For CpTi plate of the same surface area, there was measurable amount of Ti ions released into the various bio-fluids from the 1 day immersion time. The rate of release of Ti ions from CpTi sample ranged from 0.023 ± 0.002 to 0.448 ± 0.004 μg/mL/week. There was a significant difference between the amounts of Ti ions released by the CpTi sample compared to that released by TiAl_6_Nb_7_

The release of measurable amount of Al ions resulted after 12 weeks of incubation. HSCl had higher Al ions release rate (0.014 ± 0.001 μg/mL/week) compared to HSpH7 (0.007 ± 0.001 μg/mL/week). The results inferred that variations in pH and chloride ions of the test medium had a significant effect on the amounts of Ti ions released, while increase in chloride ions concentration significantly elevates the release of Al ions into the bio-fluids. The small amount of released metal ions measured in this study cannot constitute a health hazard to the host.

Notwithstanding, there is need for more long-term investigations of metal ions released from Ti and Ti based alloys into bio-fluids since patients hosts these biomaterials throughout life time, used either for dental and orthopedic replacements or as reported by studies for the systemic accumulation of metal ion in organs of post-operative patients of titanium implants and restorations.
